# Passive Electrical Components Based on Cotton Fabric Decorated with Iron Oxides Microfibers: The Influence of Static and Pulsed Magnetic Fields on the Equivalent Electrical Properties

**DOI:** 10.3390/mi14112061

**Published:** 2023-11-04

**Authors:** Ioan Bica, Eugen Mircea Anitas, Hyoung-Jin Choi, Shizhao Wang

**Affiliations:** 1 Department of Physics, West University of Timisoara, V. Parvan Avenue 4, 300223 Timisoara, Romania; ioan.bica@e-uvt.ro; 2 Department of Physics, Craiova University, A. I. Cuza Street 13, 200585 Craiova, Romania; 3 Joint Institute for Nuclear Research, 141980 Dubna, Russia; 4 Horia Hulubei, National Institute of Physics and Nuclear Engineering, 077125 Magurele, Romania; 5 Department of Polymer Science and Engineering, Inha University, Incheon 22212, Republic of Korea; hjchoi@inha.ac.kr (H.-J.C.); 22201657@inha.edu (S.W.)

**Keywords:** composite materials, cotton fibers, iron oxide microfibers, silicone oil, static magnetic field, pulsed magnetic field, complex dielectric permittivity, electrical conductivity, electric field sensor, magnetic field sensor

## Abstract

In this work, environmentally friendly and low-cost passive electrical components (PECs) are manufactured based on composites consisting of cotton fabrics soaked with solutions of silicone oil and different amounts of iron oxides microfibers (μFe). The μFe consists of a mixture of three phases: hematite (α-Fe${}_{2}$O${}_{3}$), maghemite (γ-Fe${}_{2}$O${}_{3}$), and magnetite (Fe${}_{3}$O${}_{4}$). The equivalent electrical capacitance ($C_{p}$) and resistance ($R_{p}$) of PECs are measured as a function of magnetic flux density *B* in a static and pulsed magnetic field superimposed on an alternating electric field of frequency 1 kHz. The relative variation in the hysteresis curves for both $C_{p}$ and $R_{p}$ are obtained by measuring them in the ascending and then the descending mode of *B*. We show that all these three quantities are sensibly influenced by the volume fractions of μFe and by the values of *B*. The main influence on this behavior is attributed to the semiconductor properties of the α-Fe${}_{2}$O${}_{3}$ and γ-Fe${}_{2}$O${}_{3}$ components of the oxide microfibers. In addition, it is found that at B≃ 175 mT, the maximum relative variance of the hysteresis curve is about 3.35% for $C_{p}$ and 3.18 % for $R_{p}$. When a pulsed magnetic field is used, it is shown that $C_{p}$ and $R_{p}$ closely follow the variation in the magnetic field. Thus, the resulting electrical properties of PECs, together with the fast response to the application of pulsed magnetic fields, make them useful in the fabrication of various devices, such as electric, magnetic, and deformation fields, or mechanical stress sensors with applications in protection against electromagnetic smog, healthcare monitoring, or for human–machine interfacing.

## 1. Introduction

Passive electrical components (PECs) are elements of electrical circuits consisting of laminated solid alloy materials, often drawn in the form of semiconducting wires, that function without a power source but that can dissipate or store electrical power [1,2]. The most common elements are resistors [3,4,5], with the role of delimiting the intensity of the electric current through the electric circuits; capacitors [6], with the role of storing electrical energy in direct current circuits and with the functions of storing electrical and reactive energy in alternating current circuits; and inductors [7], with the role of storing energy in a magnetic field when an electric current flows through it. Other important categories are coils, with resistive functions in direct current circuits, and resistive and reactive functions in alternating current circuits; ferrites, which can be represented as a combination of a coil, a capacitor, and an electrical resistance; potentiometers, defined as variable resistors [8]; varistors [9]; photoresistors [10]; and thermistors [11]. The later three categories are resistors whose electrical resistance is controlled, in turn, by the applied electrical voltage, the luminous flux, and the incident thermal radiation.

Relatively recently, great interest has been focused on fabrication of PECs based on polyphasic liquid solutions, also known as magnetorheological suspensions (MRSs) [12,13,14]. In general, MRSs consist of a base liquid of silicone/mineral/vegetable oil, honey, or water, in which ferro- or ferrimagnetic magnetic microparticles are dispersed together with additives such as nanofibers, magnetite nanoparticles, or guar gum [15,16,17]. When an external magnetic field is applied, the magnetic dipoles from the base liquid form chain-like aggregates oriented along the magnetic field lines [14]. Thus, the induced effects transform MRSs into an electrically and thermally conductive viscous substance [15,16,18]. This property is used in fabrication of dampers of vibrations and mechanical shocks, clutches, valves, or batteries [19].

When the intensity of the magnetic field is varied, the distances between magnetic dipoles from within the chains are also changed. Then, the three-dimensional structures of dipoles form networks of microresistors connected in series or in parallel with electrical microcapacitors [14]. Thus, overall, from an electrical point of view, MRSs are characterized by a resistor with an equivalent electrical resistance $R_{p}$, in cases when a continuous current is applied [13], and, respectively, by a resistor connected in series or in parallel with an equivalent electric capacitor of capacitance $C_{p}$, when an alternating electric current is applied [12,13]. In both cases, the values of $R_{p}$ and $C_{p}$ depend on the magnetic field intensity and on the volume fraction of the magnetizable phase inside the base liquid.

To provide a greater mechanical stability and flexibility, an increased sedimentation time, and to partially avoid clumping of the magnetizable phase, a dry matrix consisting of natural polymers of cotton fibers or polyurethane sponge soaked with a solution based on carbonyl iron (CI) microparticles is often used [20,21]. As in the case of the classical MRSs discussed above, it has been shown that for MRSs reinforced with natural fibers, the values of $R_{p}$ and $C_{p}$ can be still significantly tuned when subjected to application of a static electric and magnetic field or to a magnetic field superimposed on a mechanical deformation field. However, when only a static electric field is applied, the electrical properties of these MRSs are not significantly changed [12,13]. Instead, installation of the final values of $R_{p}$ and $C_{p}$ is realized with a significant delay. Moreover, the surface area of the hysteresis curves of $R_{p}$ and $C_{p}$, measured as a function of magnetic flux density *B*, is quite pronounced and depends on the volume fraction of CI microparticles.

In the realm of material science for PECs, the use of cotton fabric decorated with iron oxide microfibers (μFe) offers several distinct advantages over conventional materials. First, the natural porosity and fibrous structure of cotton provide an excellent scaffold for μFe, enhancing the mechanical integrity and durability of the composite material [22,23]. This synergistic combination results in a material with superior mechanical properties, including tensile strength and flexibility, which are crucial for applications requiring mechanical robustness. Second, μFe contributes to the material’s magnetic properties, thereby allowing for more precise control of electrical resistance and capacitance when subjected to magnetic fields [14]. Moreover, in light of relatively recent research on the rheology of magnetic fiber suspensions, which reveals that fiber-based aggregates exhibit a dynamic yield stress three times higher than their spherical particle counterparts under certain magnetic field intensities [24], the use of μFe may offer additional advantages in terms of mechanical stability and tunable magnetic properties. This is particularly beneficial in applications requiring rapid response times and high sensitivity to external stimuli. Furthermore, the biocompatibility of cotton makes these composites particularly attractive for biomedical applications, such as in the development of smart prosthetics or bio-sensors [25,26].

In order to obtain PECs with a fast response, with small hysteresis curves for $R_{p}$ and $C_{p}$, and whose values can be sensibly changed in a continuous/pulsed magnetic field superimposed on an alternating electric field, in this work, we manufacture three PECs having as dielectric material composites based on cotton fibers soaked with MRS solutions of silicone oil (SO) and μFe at different concentrations. To this aim, we present the materials used for preparation of the composite in Section 2.1, and the main structural, elemental and magnetic characterisation of μFe in Section 2.2. This is followed by Section 3.1, which shows the main steps in preparation of MRSs together with their magnetic properties. In Section 3.2, the main steps in the preparation of PECs are presented, and in Section 3.3, an in-house overall setup used to investigate the electrical properties of PECs is described. Section 3.4 and Section 3.5 present detailed results and discussions concerning the electrical resistance and capacitance of PECs in a static, and, respectively, pulsed magnetic field, Section 3.6 shows the hysteresis curves for $R_{p}$ and $C_{p}$ and, finally, Section 3.7 presents results concerning complex dielectric permittivity, magnetodielectric and magnetoconductive effects, as well as basic concepts of dielectric theory needed to understand the physical mechanisms that lead to the observed effects.

## 2. Materials and Methods

### 2.1. Raw Materials

The materials used for fabrication of the composite materials are: gauze bandage (GB) from Medicomp Hartmann (Bucharest, Romania), in the form of textile fabric, based on white cotton fibers (predominantly composed of cellulose, about 98%, and minor fractions of hemicellulose and lignin with ≲1% for each polymer type [27]) with a thickness of 0.50 mm and a granulation of 30 g/cm${}^{2}$; SO, MS100 type from Siliconi Commerciale SpA (Gambellara-Vicenza, Italy), with a density of 0.97 g/cm${}^{3}$ and dynamic viscosity 97 mPa·s at 25 °C; and μFe obtained my microwave synthesis [28] of 2 cm${}^{3}$ of CI microparticles form Sigma-Aldrich (Taufkirchen, Germany), 5 cm${}^{3}$ of SO, and 1 cm${}^{3}$ of iron pentacarbonyl, also from Sigma-Aldrich (Darmstadt, Germany). Their mass density is 2.88 g/cm${}^{3}$.

### 2.2. Structural, Elemental, and Magnetic Characterization of Iron Oxide Microfibers

A scanning electron microscope (SEM) Inspect S PANalytical model from FEI Company (Eindhoven, The Netherlands) was used in low-vacuum mode using an LFD detector, spot value of 4, and high voltage (HV) of 30.00 kV, coupled with the energy dispersive X-ray (EDX) analysis detector with energies generated up to about 12 keV, was used to characterize the surface morphology of the fibers, using catalyst powder supported on carbon tape, as described in Ref. [28].

**Figure 1:**
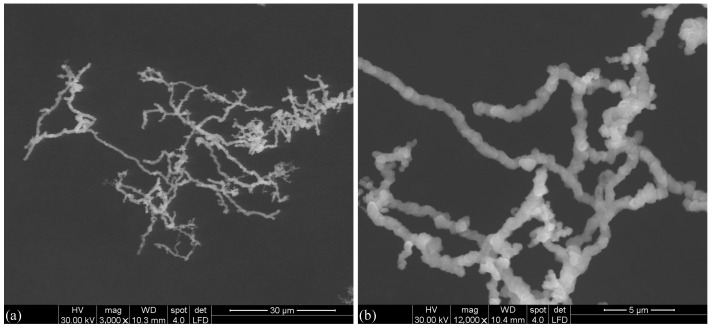
SEM images of μFe at two different magnifications. (**a**) 3000×. (**b**) 12,000×.

**Figure A1:**
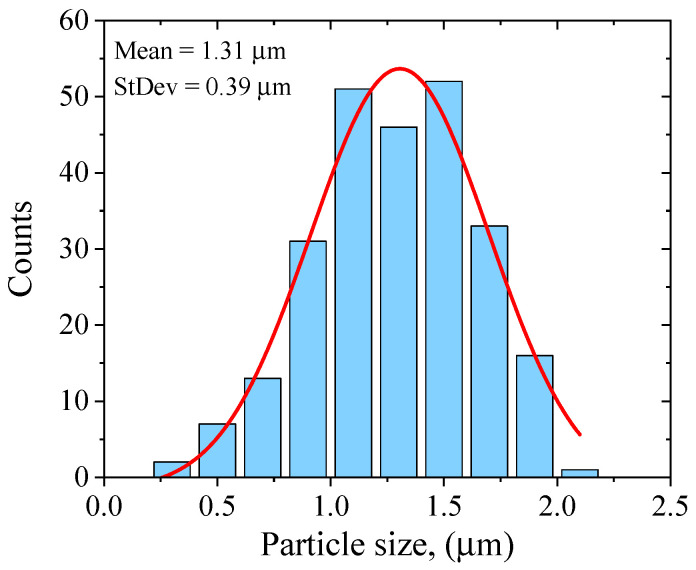
Histogram of size distribution of 250 particles forming the μFe (light-blue columns) and a fit with a Gaussian distribution (red continuous curve).

The results are presented in Figure 1a,b at two different magnifications, and show that the μFe diameters range between 0.25 μm and 2.20 μm, and they have lengths of several tens of micrometers. The basic components of these microfibers are particles with a mean diameter of about 1.31 μm and standard deviation of about 0.39 μm (see Figure A1 in Appendix A for more details). At a larger scale, the μFe forms a fractal structure with fractal dimension of about 1.82 [28]. This corresponds to a complex morphology consisting of a relatively close structure (the closer the fractal dimension to 2.00 the more branched the structure is, and vice versa). Therefore, such fibers are promising candidates to be used as additives for fabrication of MRSs with good anti-sedimentation properties.

**Figure 2:**
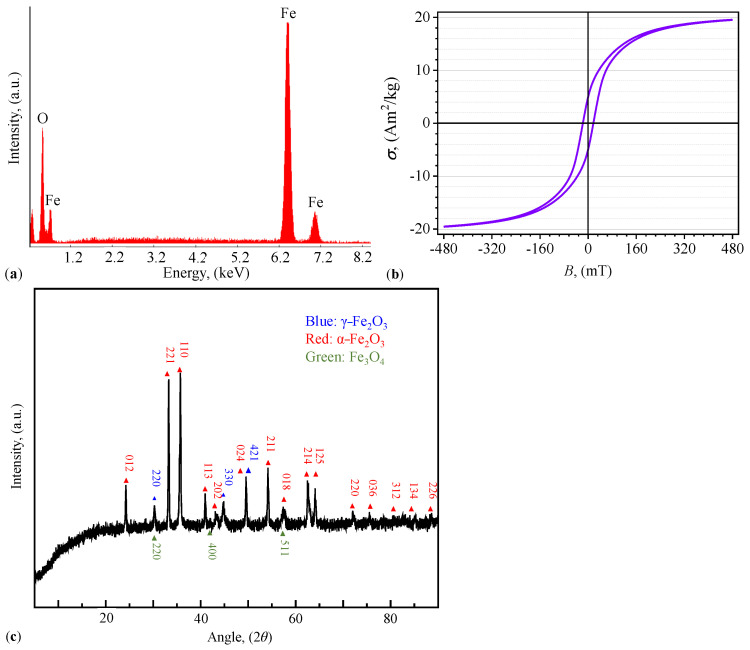
Elemental composition, magnetic properties, and crystallographic structure of μFe. (**a**) EDX spectrum. (**b**) Specific magnetization σ as a function of magnetic flux density *B*. (**c**) XRD spectrum.

**Figure A2:**
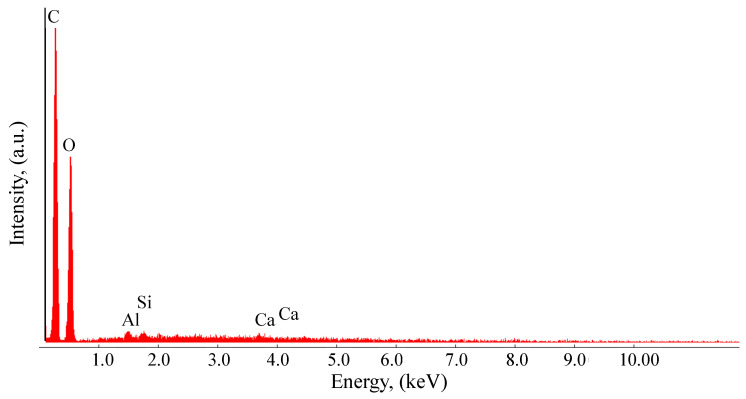
Elemental analysis of cotton fibers. C—59.97% wt., O—41.30% wt., Al—0.32% wt., Si—0.18% wt., Ca—0.22% wt.

Elemental analysis is presented in Figure 2a for energies up to 8.2 keV. For higher energies, no additional signal was recorded. The results show that the fibers consist of iron oxides and reveals the presence of oxygen and iron at two different mass ratio that correspond to ferric (Fe${}_{2}$O${}_{3}$) and magnetite (Fe${}_{3}$O${}_{4}$) oxides [28]. The main contribution to the magnetic properties is given by the later oxide. Elemental analysis for the pure cotton fibers show that they are composed mostly of C and O (see Figure A2 in Appendix B).

The magnetization curve of μFe is shown in Figure 2b, and it has been obtained by means of a laboratory-made alternating current induction hysteresis graph developed in Ref. [29]. The accuracy of the instrument is 1.5% of the full scale. There are several reasons for adopting this method here. First, sample geometry and material composition: the vibrating sample magnetometer technique [30] poses challenges for our specific sample types, which consist of either powder or viscous magnetic composites. Utilizing a vibrating sample magnetometer could potentially alter the intrinsic structure of these materials, thereby compromising the integrity of our results. Second, field strength capability, as discussed in the user manual provided by the manufacturer of the AC magnetic susceptibility technique described in Ref. [31]. Third, apart from calibration difficulties (in terms of magnetization or specific magnetization units), for reasons regarding the available amount of sample material (sufficient in our case), magnetic force microscopy [32] does not seems to be justified. For the samples used here, the specific saturation magnetization is $\sigma_{s}=19.5$ Am${}^{2}$/kg at B≳477 mT. Additional details are given in Ref. [28].

Identification of the crystallographic phase was performed on data shown in Figure 2c, which was obtained by using X-ray diffraction with a Rigaku DMAX-2500 diffractometer (Tokyo, Japan), Cu-Kα radiation (λ=0.15406 nm), and 2θ ranging from 20° to 80° [28]. Here, by applying Rietveld refinement method using JADE software version number 9.0.0.0 [33], it is found that μFe consists of hematite (α-Fe${}_{2}$O${}_{3}$), maghemite (γ-Fe${}_{2}$O${}_{3}$), and Fe${}_{3}$O${}_{4}$ oxides, with relative percentages of 17.80%, 51.50%, and 30.70%, respectively.

## 3. Results and Discussion

### 3.1. Preparation, Structural and Magnetic Properties of MRSs

**Figure 3:**
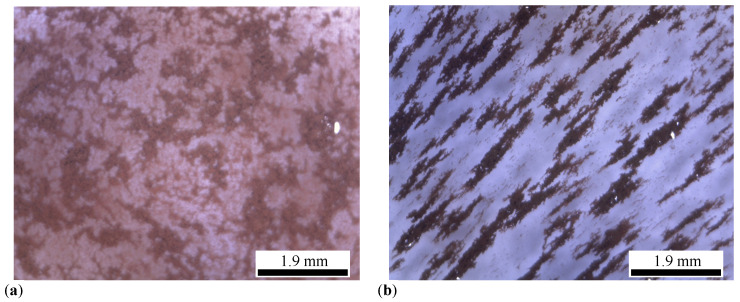
Optical microscopy images of solution L${}_{1}$. (**a**) Without a magnetic field. (**b**) With a magnetic field of flux density B≃50 mT.

**Figure 4:**
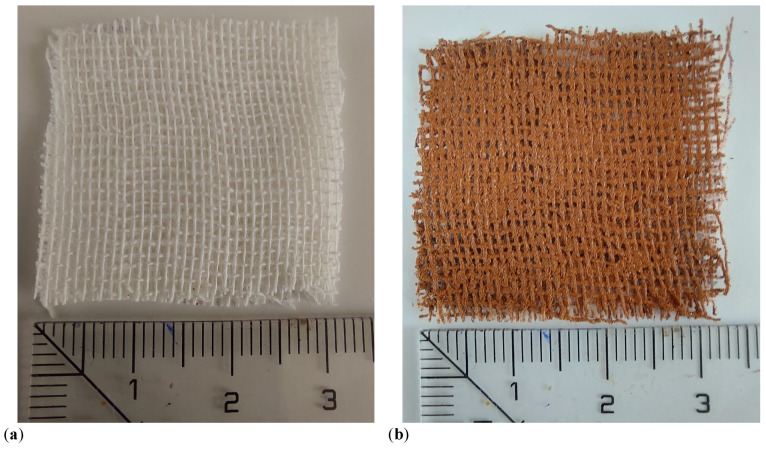
Photos of GB (**a**) and resulting MRSs-based composite (**b**). The units of the values marked on the ruler are in cm.

The main steps followed in preparation of MRSs are:

1. Three different masses *m* of μFe and SO are weighed, having the values listed in Table 1. Then, they are mixed by turn in Berzelius glasses and biphasic liquids are formed.
2. Each liquid mixture is homogenized for about 300 s at temperatures ranging from 150 °C to 180 °C. After this time period, the temperature of the mixture is allowed to decrease and to reach the room temperature. During this cooling time the mixture is still homogenized. At the end of this step, one obtains the MRSs${}_{i},i=1,2,3$, with the mass fractions $\Phi_{\mu \mathrm{Fe}}$ (wt.%) and $\Phi_{\mathrm{SO}}$ (wt.%) listed in Table 1. Figure 3a,b shows images taken with a BPM-350 Digital Microscope for Industrial Inspection, of the solution L${}_{1}$ without, and with an applied magnetic field of flux density B≃50 mT, respectively. In the later case, μFe form chain-like aggregates oriented along the magnetic field lines, as also shown previously [12,14]. This effect is responsible for the properties of PECs (see Section 3).
3. Three fabrics with dimensions 30 mm × 30 mm × 0.5 mm are cut from GB. Figure 4a shows an image of a single piece.
4. The first GB fabric is soaked with a random volume of 0.8 cm${}^{3}$ taken from solution L${}_{1}$, and the MRSs${}_{1}$-based composite is obtained. Similar steps are repeated for the second and third fabric, in order to obtain the composites based on MRSs${}_{2}$ and MRSs${}_{3}$. The obtained composites have a dark color, as shown in Figure 4b.

**Figure 5:**
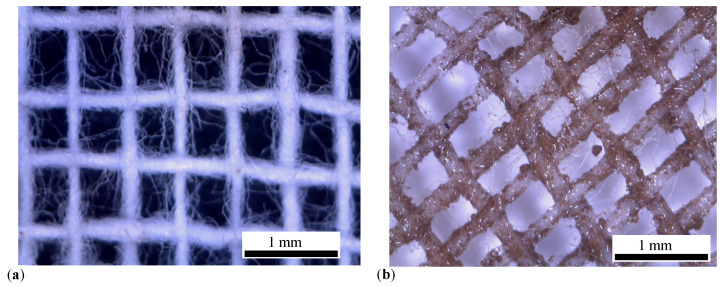
Threads of GB cotton fabrics visualized with the optical microscope. (**a**) Before soaking with liquid solutions L${}_{i}$. (**b**) After soaking with liquid solutions L${}_{i}$. Brownish regions—agglomerates of μFe. White spots on the cotton threads—light reflections.

Images taken with the same optical microscope reveal that GB is formed of interwoven threads forming knots and stitches (see Figure 5a). Each thread is made of spaced microfibers. By soaking them with the liquid solutions L${}_{i},i=1,2,3$, the cotton fibers absorb the μFe through capillarity (see Figure 5a), thus becoming magnetizable.

For obtaining the magnetization curves of the MRSs${}_{i},i=1,2,3$ we take into account that between the saturation magnetization of μFe ($\sigma_{s}$) and the magnetization of MRSs${}_{i}$ ($\sigma_{{\mathrm{MRSs}}_{i}}$), the following relation holds [34]:

(1) $$\mu_{0}\sigma_{{\mathrm{MRSs}}_{i}}=\left(\rho_{\mathrm{SO}}/\rho_{\mu \mathrm{Fe}}\right)\Phi_{\mu {\mathrm{Fe}}_{i}}\mu_{0}\sigma_{s},i=1,2,3,$$

where $\mu_{0}$ is the vacuum magnetic constant, $\rho_{\mathrm{SO}}$ is the density of SO, $\rho_{\mu \mathrm{Fe}}$ is the density of μFe, and $\Phi_{\mu {\mathrm{Fe}}_{i}}$ is the mass fraction of μFe.

Therefore, by using the above data for the density of SO and μFe in Equation (1) one can see that the saturation magnetizations of MRSs${}_{i}$ are similar, up to a constant shift (see Table 1), to that of the μFe shown in Figure 2b. However, the values of specific magnetization are sensibly influenced by the values of mass fractions of μFe from within the SO.

### 3.2. Fabrication of PECs

**Figure 6:**
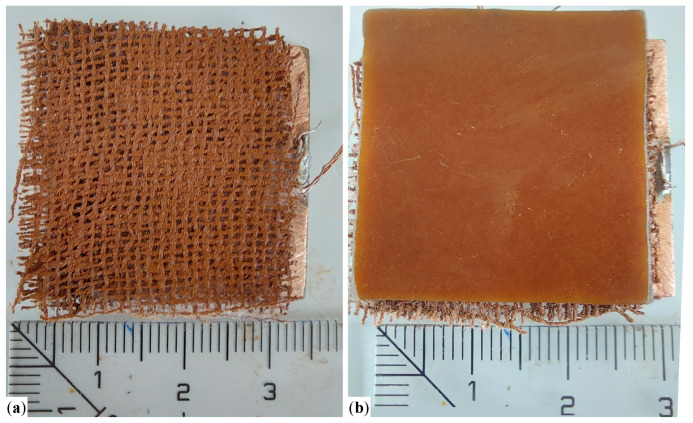
Image of MRSs_1_ on top of the copper-side of a textolite plate (**a**), and of MRSs_1_ between the copper-sides of two textolite plates, i.e., PEC${}_{1}$ (**b**). The units of the values marked on the ruler are in cm.

The main steps in fabrication of PECs are the following:

1. Six plates of dimensions 30 mm × 30 mm are debited from a simple sticlotextolite having one side covered with a copper foil;
2. On the copper side of the textolite, MRSs${}_{1}$-based composite is deposited and a subassembly is obtained, as shown in Figure 6a;
3. A second textolite plate is used to cover the subassembly obtained at step 2, with the copper side touching the MRSs${}_{1}$. At the end of this step, one obtains PEC${}_{1}$, as shown in Figure 6b;
4. In order to obtain PEC${}_{1}$ and PEC${}_{2}$, steps 2 and 3 are repeated by using MRSs${}_{2}$, and, respectively, MRSs${}_{3}$-based composites.

### 3.3. Experimental Setup for Studying the Effects of the Magnetic Field on PECs

**Figure 7:**
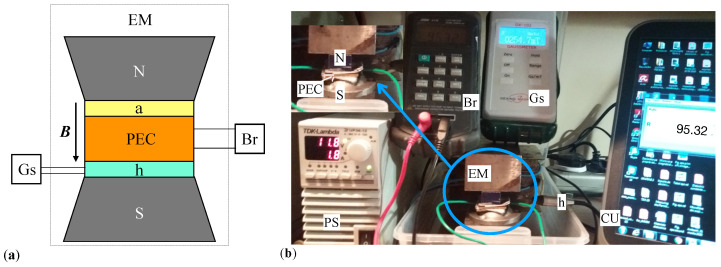
Experimental setup for studying the effects of magnetic field on PECs. (**a**) Overall configuration. (**b**) Photo of the whole setup. EM—electromagnet, PS—power source, Br—RLC bridge, Gs—Gaussmeter, h—Hall probe, CU—computing unit, N and S—magnetic poles, PEC—passive electric component, ***B***—magnetic flux density vector, a—nonmagnetic disk for fixation of PEC with h. The size of EM encircled in the blue ellipse is slightly increased and shown on the upper-left corner, as indicated by the blue arrow.

A schematic representation of the experimental setup used for investigating the magnetodielectric effects, the response speed of PECs, and the hysteresis effects induced by an external magnetic field on $R_{p}$ and $C_{p}$ of PECs is shown in Figure 7a. It consists of an electromagnet EM, a power source PS, a bridge Br (type E7-20, MNIPI, Minsk, Belarus), a Gaussmeter Gs (type DX-102, Dexing Magnet Tech. Co., Xiamen, China), and a computing unit CU with software for data acquisition and analysis received from Br.

An image of the whole setup is shown in Figure 7b. The electromagnet is an in-house built device with the following parameters in a continuous current mode: electrical resistance $R_{\mathrm{EM}}=6.49\;\Omega$, inductance $L_{\mathrm{EM}}=0.16$ mH and electrical capacitance $C_{\mathrm{EM}}=1.12$ mF. Between the north (N) and south (S) poles of the electromagnet PEC and the Hall probe h of Gs is fixed. The distance between poles is adjustable.

The power source (type TDK-Lambda, TDK-Corporation, National City, CA, USA), allows to maintain a constant intensity of the electric current through the coil of EM. Through its control elements, it is possible to continuously adjust the intensity of the electric current up to a maximum of 12 A, or in pulses with the repetition period and the filling factor of the intensity of the electric current through the coil of EM. During measurements, the bridge Br is set on measuring the parallel electrical components of PECs, at a frequency of f= 1 kHz and effective voltage U=1 V. The Gaussmeter Gs allows a continuous reading of the magnetic flux density through the Hall probe h, fixed under PECs, between N and S poles of EM.

### 3.4. Equivalent Electrical Capacitance and Resistance of PECs in a Static Magnetic Field

The experimental setup described above is used as follows: first, a volume of 0.8 cm${}^{3}$ of SO is soaked into GB (Figure 4a). The resulting composite is placed between the copper-sides of textolite plates. As such, one obtains a reference PEC, i.e., a PEC without μFe, but with the geometry of PEC${}_{i},i=1,2,3$ shown in Figure 6b. By using the RLC bridge Br shown in Figure 7b, one measures $C_{p}$ and $R_{p}$ of the reference PEC and obtains $C_{p0}=41.5$ pF and $R_{p0}=240$ kΩ. Since the reference PEC does not contain a magnetizable phase, the values of $C_{p0}$ and $R_{p0}$ remain unchanged with the application of a magnetic field.

**Figure 8:**
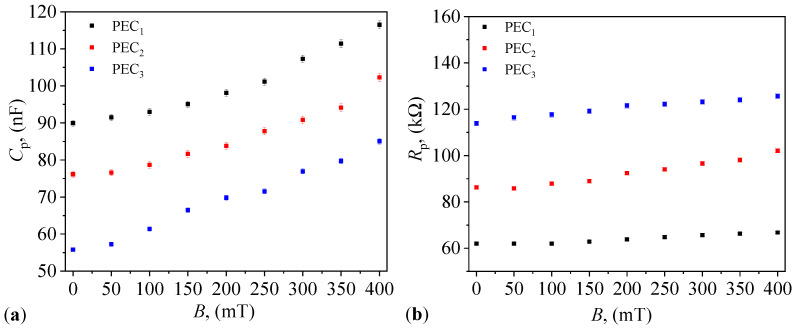
Variation in the equivalent electrical capacitance $C_{p}$ (**a**) and resistance $R_{p}$ (**b**) with the magnetic flux density *B* for the three PECs in the static magnetic field.

Second, PEC${}_{i},i=1,2,3$ are fixed, in turn, together with the Hall probe h, between N and S poles of EM, as shown in Figure 7b, and are pressed until the thickness of the composites becomes equal to that of GB (i.e., 0.5 mm). The thickness is controlled with the help of a non-magnetizable tape and remains unchanged when a magnetic field is applied. By using the same RLC bridge as above, one measures 12 times $C_{p}$ and $R_{p}$ for all three PECs in a magnetic field with flux density *B* varying from 0 to 400 mT in steps of 50 mT. Data for each individual measurement are given in Table A1, Table A2, Table A3, Table A4, Table A5 and Table A6 in Appendix C. The average values together with the standard deviations are presented in Figure 8a, and, respectively, Figure 8b.

The results show that for fixed values of magnetic flux density *B*, the capacitance $C_{p}$ decreases, and resistance $R_{p}$ increases with mass fraction of μFe. However, for a fixed mass fraction of μFe, both $C_{p}$ and $R_{p}$ increase with *B*. Such a behavior is specific to PECs based on composite materials [35], and is different as compared to PECs based on cotton fabrics soaked with CI microparticles, and where $C_{p}$ increases and $R_{p}$ decreases with increasing the mass fraction of CI [20,21]. This difference arise due to the interfacial polarization properties of γ-Fe${}_{2}$O${}_{3}$ and Fe${}_{3}$O${}_{4}$ oxides [36,37], due to the dielectric heterogeneities of the μFe/cotton fibers/copper foils complex [35,38], and to the semiconducting properties of α-Fe${}_{2}$O${}_{3}$ [36] and γ-Fe${}_{2}$O${}_{3}$ [39] oxides. The induced polarization effects relate to the accumulation of charges that creates a localized electric field which opposes the external electric field (of frequency *f* = 1 kHz and effective intensity E≡U/h=1V/0.5mm=2 kV/m, where *U* is the effective voltage). Thus, the movement of free charges is prevented by the components of hMRSs by formation of separation zones between different dielectric components. Note that for 200mT≲B≲400mT, both $C_{p}$ and $R_{p}$ have a quasi-linear linear increase, thus making further increases in the *B* less informative for the specific envisioned applications. In particular, for protection of human personnel against electromagnetic smog, relatively low values of *B* (up to about 400 mT) are generally sufficient.

### 3.5. Equivalent Electrical Capacitance and Resistance of PECs in a Pulsed Magnetic Field

In order to achieve a step-like magnetic field, the intensity of electric current passing through the coil of EM is fixed (from the power source PS) such that the periodicity is 120 s. We consider that at t=0 s, B=200 mT. At the terminals of PECs, fixed by turn between N and S poles of EM, one measures (through the RS-232C interface) the values of $C_{p}$ and $R_{p}$. As such, we obtain the variation in capacitance and resistance with time at fixed mass fractions of μFe, i.e., $C_{p}=C_{p}{\left(t\right)}_{\Phi_{\mu \mathrm{Fe}}}$ and $R_{p}=R_{p}{\left(t\right)}_{\Phi_{\mu \mathrm{Fe}}}$.

**Figure 9:**
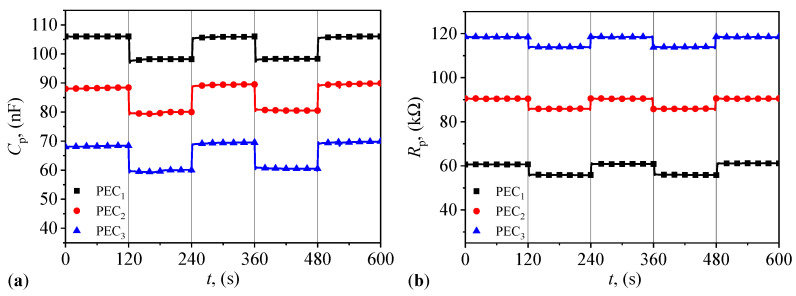
Variation in the equivalent electrical capacitance $C_{p}$ (**a**) and resistance $R_{p}$ (**b**) with the magnetic flux density *B* for the three PECs in a pulsed magnetic field (see main text for details) measured with a CHY 41R RLC meter from Centenary Materials Co. (Hsinchu, Taiwan). Dots—experimental data. Continuous lines—spline interpolation.

The results are presented in Figure 9a and Figure 9b, respectively, and they clearly indicate that the response functions of PECs are also step-like for each mass fraction of μFe. The average values of $C_{p}$ and $R_{p}$ and their standard deviations, for each time interval with a 120 s periodicity, are reported in Table A13 and Table A14 in Appendix D, respectively. The results show very small standard deviations for each interval. Due to the low mass density of μFe, the values of $C_{p}$ and $R_{p}$ are instantaneously installed and closely follow the step-like structure of magnetic field. The vertical lines in Figure 9a,b underline this behavior. This effect is similar to the one obtained in hybrid MRSs or in magnetoactive elastomers comprising micrometer-sized iron particles dispersed in compliant elastomer matrices [40].

### 3.6. Hysteresis Curves of Equivalent Electrical Capacitance and Resistance of PECs in a Static Magnetic Field

**Figure 10:**
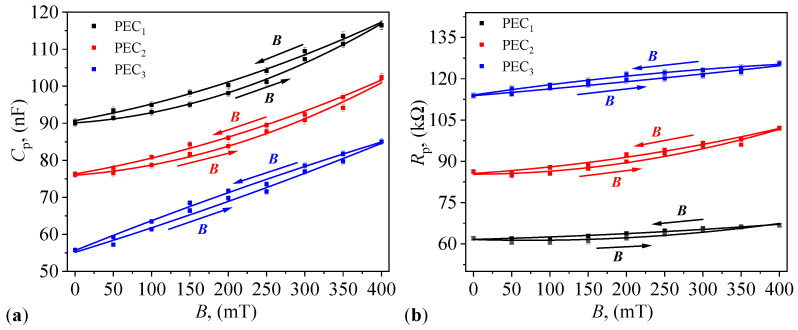
Variation in the equivalent electrical capacitance $C_{p}$ (**a**) and resistance $R_{p}$ (**b**) with the magnetic flux density *B* for the three PECs, in the static magnetic field and with increasing and decreasing values of magnetic flux density *B*. Dots—experimental data. Continuous lines—polynomial fit.

The values of equivalent electrical capacitance $C_{p}$ and resistance $R_{p}$ of PECs are recorded in a static magnetic field when the magnetic flux density *B* is first increased and then decreased. The step used is 50 mT at a time interval of 5 s. The graphical representation of average values and corresponding standard deviations is shown in Figure 10a and Figure 10b, respectively. Data for each individual measurement are given in Table A1, Table A2, Table A3, Table A4, Table A5, Table A6, Table A7, Table A8, Table A9, Table A10, Table A11 and Table A12 in Appendix C. The results indicate that the variation in $C_{p}$ and $R_{p}$ is characterized by hysteresis curves with a small surface area. To explain this, note from Figure 2 that when *B* is decreased, the values of σ are higher as compared to the case when *B* is increased, for a fixed *B*. This leads to an increase in the interfacial electrical charges and a decrease in the electric leakage current through PECs. The net effect is an increase in $C_{p}$ and $R_{p}$ when *B* is decreased, and thus the formation of the observed hysteresis curves.

**Figure 11:**
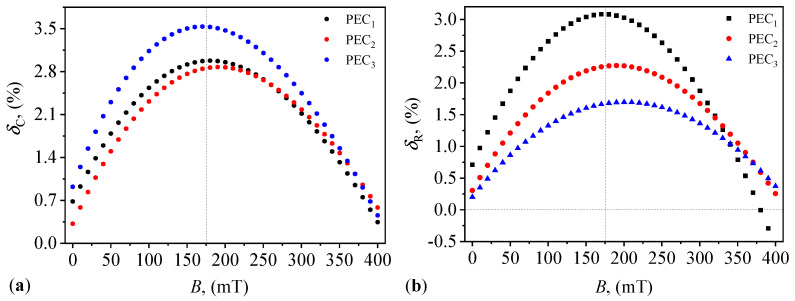
Relative variation in the width of hysteresis curves for electrical capacitance $C_{p}$ (**a**) and resistance $R_{p}$ (**b**) for the three PECs, with magnetic flux density *B*. Vertical dashed lines—position of maxima for PEC${}_{1}$.

The relative variation in the width of the hysteresis curves for $C_{p}$ is defined as:

(2) $$\delta_{C}\left(\%\right)=\left(\frac{C_{p↓}}{C_{p↑}}-1\right)\times 100,$$

where $C_{p↑}$ is the value of $C_{p}$ when *B* is increased, and $C_{p↓}$ is the value of $C_{p}$ when *B* is decreasing. Then, by using the variation in $C_{p↑}$ and $C_{p↓}$ with *B* at constant mass fractions $\Phi_{\mu Fe}$, i.e., $C_{p↑}=C_{p↑}{\left(B\right)}_{\Phi_{\mu \mathrm{Fe}}}$ and $C_{p↓}=C_{p↓}{\left(B\right)}_{\Phi_{\mu \mathrm{Fe}}}$ from Figure 10a in Equation (2), one obtains the variation $\delta_{C}=\delta_{C}{\left(B\right)}_{\Phi_{\mu \mathrm{Fe}}}$ as shown in Figure 11a.

The results indicate that for each PEC, the values of $\delta_{C}$ are positive in the whole range of *B* and the maximum relative variation in the hysteresis curve is less than 3.5% (at B≃175 mT, as indicated by the vertical dashed line). This corresponds to a difference of 0.44 Am${}^{2}$/kg in specific magnetization of μFe (see Figure 2). In addition, for PEC${}_{1}$ and PEC${}_{2}$, the values of $\delta_{C}$ are quite close, while for PEC${}_{3}$, they are clearly distinct. Such differences for PEC${}_{3}$ arise due to the relative high concentration of γ-Fe${}_{2}$O${}_{3}$ and Fe${}_{3}$O${}_{4}$ oxides. Similar changes in $\delta_{C}$ with increasing the quantity of magnetizable phases, and for different types of matrices were also reported in Refs. [37,40]. Therefore, due to this high precision of $\delta_{C}$, the obtained PECs have good properties for practical applications, such as sensors, transducers, or electromagnetic absorbers.

Similarly, the relative variation in the width of hysteresis curves for $R_{p}$ is defined as:

(3) $$\delta_{R}\left(\%\right)=\left(\frac{R_{p↓}}{R_{p↑}}-1\right)\times 100,$$

where $R_{p↑}$ is the value of $R_{p}$ when *B* is increased, and $R_{p↓}$ is the value of $R_{p}$ when *B* is decreasing. Then, by using the variation in $R_{p↑}$ and $R_{p↓}$ with *B* at constant mass fractions $\Phi_{\mu Fe}$, i.e., $R_{p↑}=R_{p↑}{\left(B\right)}_{\Phi_{\mu \mathrm{Fe}}}$ and $R_{p↓}=R_{p↓}{\left(B\right)}_{\Phi_{\mu \mathrm{Fe}}}$ from Figure 10a in Equation (2), one obtains the variation $\delta_{R}=\delta_{R}{\left(B\right)}_{\Phi_{\mu \mathrm{Fe}}}$ as shown in Figure 11b. As in the case of $\delta_{C}$, $\delta_{R}$ has a maximum value of only 3% at B≃175 mT for PEC${}_{1}$ (indicated by the vertical dashed line). In addition, at B≳375 mT, $\delta_{R}$ for all PECs is less that 0.5%, and therefore for practical applications that require magnetic field measurements, one can use the equivalent resistance instead of capacitance for even higher precision.

### 3.7. Complex Dielectric Permittivity of PECs

The results shown in Figure 8 suggest that PECs can be represented as a plane electrical capacitor connected in parallel to a linear electrical resistor. Then, the capacitance $C_{p}$ and resistance $R_{p}$ of PECs can be obtained from

(4) $$C_{p}=\epsilon_{0}\epsilon_{r}^{{}^{\prime }}L^{2}/h,$$

and, respectively, from

(5) $$R_{p}=\rho L^{2}/h,$$

where $\epsilon_{0}=8.854\times 10^{-7}$ F/m is vacuum dielectric constant, $\epsilon_{r}^{{}^{\prime }}$ is the relative dielectric permittivity, L=30 mm is the side length of the PECs, *h* is the thickness of the MRSs-based composite, and ρ is their electrical resistivity. Therefore, using these numerical values, the above equations can be rewritten as a function of $C_{p}$, and, respectively, of $R_{p}$, i.e.,

(6) $$\epsilon_{r}^{{}^{\prime }}=62.75\times C_{p}\left(nF\right),$$

and

(7) $$\rho =1800\times R_{p}\left(k\Omega \right).$$

It is well-known from the theory of linear dielectrics that the following relation holds between the electrical resistivity and dielectric loss factor $\epsilon_{r}^{{}^{\prime \prime }}$ [41]:

(8) $$\rho^{-1}=2\pi f\epsilon_{0}\epsilon_{r}^{{}^{\prime \prime }}.$$

Thus, by using f=1 kHz together with Equation (7) in Equation (8), one obtains the loss factor in the following form:

(9) $$\epsilon_{r}^{{}^{\prime \prime }}=9986\times R_{p}{\left(k\Omega \right)}^{-1}.$$

**Figure 12:**
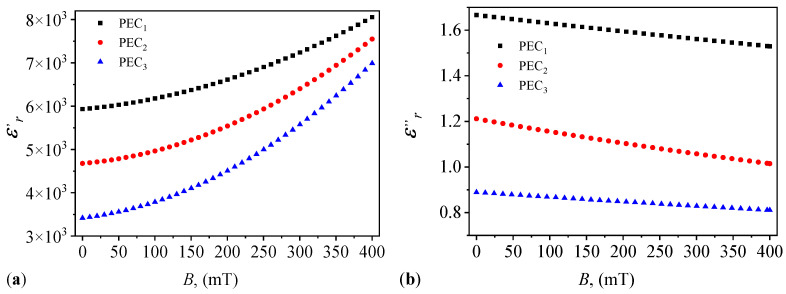
Variation in the relative dielectric permittivity $\epsilon_{r}^{{}^{\prime }}$ (**a**) and loss factor $\epsilon_{r}^{{}^{\prime \prime }}$ (**b**) with the magnetic flux density *B* for the three PECs.

Finally, by introducing the variation in capacitance from Figure 8a in Equation (6), and the variation in resistance from Figure 8b in Equation (9), one obtains the variations $\epsilon_{r}^{{}^{\prime }}=\epsilon_{r}^{{}^{\prime }}{\left(B\right)}_{\Phi_{\mu \mathrm{Fe}}}$ and $\epsilon_{r}^{{}^{\prime \prime }}=\epsilon_{r}^{{}^{\prime \prime }}{\left(B\right)}_{\Phi_{\mu \mathrm{Fe}}}$ as shown in Figure 12a and Figure 12b, respectively.

The results show that both $\epsilon_{r}^{{}^{\prime }}$ and $\epsilon_{r}^{{}^{\prime \prime }}$ are sensibly influenced by the magnetic flux density *B* and mass fraction of μFe. Such a behavior can be attributed to the coexistence of α-Fe${}_{2}$O${}_{3}$ oxide together with γ-Fe${}_{2}$O${}_{3}$ and Fe${}_{3}$O${}_{4}$ oxides. It is known that the former oxide has semiconducting properties with a width of the forbidden band gap of about 2.1 eV [36], while for γ-Fe${}_{2}$O${}_{3}$ oxide, the gap is about 4.2 eV [39]. Taking into account that during the measurements, the effective voltage is U=1 V and the frequency is f=1 kHz, then the total energy supplied to the composites between electrodes of PECs is $\epsilon \equiv C_{p}U/2$, where $54≲C_{p}\left(\mathrm{nF}\right)≲115$. By using the above numerical value of *U*, one obtains that $169≲10^{-6}\epsilon \left(\mathrm{keV}\right)≲363$, and thus the alternating electric field, debited by the source of the Br bridge, is sufficient to provide the necessary energy for the polarization process and for the electrons to pass the forbidden band and, through collisions with the atoms in the mixture, to ionize them. The obtained effect is the increase in $\epsilon_{r}^{{}^{\prime }}$ with *B*, as shown in Figure 12a. Instead, increasing the mass concentration of μFe has the effect of increasing the band gap of the semiconductor. At the same electrical energy debited by the current source of the Br bridge, the density of electrons that cross the forbidden band of the semiconductor decreases with the increase in the μFe mass fraction. The obtained effect is a decrease in the electrical conductivity, and thus of the loss factor $\epsilon_{r}^{{}^{\prime \prime }}$ of the PECs as shown in Figure 12b, together with the accumulation of electric charges at the border regions between the constituents of the MRSs-based composites.

The data in Figure 12 allows us to quantify the influence of μFe and of the magnetic field on the dielectric and conductive properties of PECs. To this aim, one can introduce the following expressions for the magnetodielectric and magnetoconductive effects:

(10) $$\alpha =\epsilon_{r}^{{}^{\prime }}/\epsilon_{r0}^{{}^{\prime }},$$

and, respectively:

(11) $$\beta =\epsilon_{r}^{{}^{\prime \prime }}/\epsilon_{r0}^{{}^{\prime \prime }},$$

where $\epsilon_{r}^{{}^{\prime }}=\epsilon_{r}^{{}^{\prime }}{\left(B\right)}_{\mu \mathrm{Fe}}$ and $\epsilon_{r}^{{}^{\prime \prime }}=\epsilon_{r}^{{}^{\prime \prime }}{\left(B\right)}_{\mu \mathrm{Fe}}$ are given in Figure 12a and Figure 12b, respectively. The quantities $\epsilon_{r0}^{{}^{\prime }}$ and $\epsilon_{r0}^{{}^{\prime \prime }}$ are the relative dielectric permittivity and the dielectric loss factor of the reference PEC described in Section 3.4, respectively, with $C_{p}0=41.5$ pF and $R_{p0}=240$ kΩ. Then, by introducing these values in Equations (6) and (9), respectively, one obtains $\epsilon_{r}^{{}^{\prime }}0=2.83$, and $\epsilon_{r}^{{}^{\prime \prime }}0=0.42$, respectively. Therefore, the curves of magnetodielectric and magnetoconductive effects given by Equations (10) and (11) are identical up to a factor of $1/\epsilon_{r}^{{}^{\prime }}0$, and$1/\epsilon_{r0}^{{}^{\prime \prime }}$ to the relative dielectric permittivity and dielectric loss factor in Figure 12, respectively. This shows that both α and β are sensibly influenced by the mass fraction of μFe and the magnetic flux density *B*.

Thus, one can choose the values of $\epsilon_{r}^{{}^{\prime }}$ and $\epsilon_{r}^{{}^{\prime \prime }}$ by fixing the mass fraction μFe and magnetic flux density *B*, such that one obtains an equivalent electrical capacitance $C_{p}$ and resistance $R_{p}$ with required values for various practical applications of PECs.

## 4. Conclusions

In this study, the manufacturing process and electrical properties of a new class of ecological and low-cost passive electrical components in the presence of static and pulsed magnetic fields has been presented in detail. The components are realized from a mechanically flexible composite material based on cotton fabric soaked with a mixture of silicone oil and iron oxide microfibers μFe, placed between two parallel textolite copper plates (Figure 6b). The mass fraction $\Phi_{\mu \mathrm{Fe}}$ of μFe in the mixture is 2 wt.%, 4 wt.%, and 6 wt.%.

The electrical properties are investigated by using an in-house built experimental setup (Figure 7b) which reveals that each obtained component can be assimilated to a plane capacitor (of capacitance $C_{p}$) connected in parallel to a linear resistor (of resistance $R_{p}$).

It is shown that in the presence of a static magnetic field, both $C_{p}$ and $R_{p}$ are sensibly influenced by $\Phi_{\mu \mathrm{Fe}}$ and magnetic flux density *B* (Figure 8). In particular, at fixed $\Phi_{\mu \mathrm{Fe}}$, $C_{p}$ and $R_{p}$ increase with *B*. However, at fixed *B*, $C_{p}$ decreases with $\Phi_{\mu \mathrm{Fe}}$, and $R_{p}$ increases with $\Phi_{\mu \mathrm{Fe}}$. As a consequence, the relative dielectric permittivity and dielectric loss factor have also a behavior sensibly influenced by $\Phi_{\mu \mathrm{Fe}}$ and *B* (Figure 12). This is explained through the semiconducting properties of α-Fe${}_{2}$O${}_{3}$ oxide from within μFe, together with the contributions resulting from interfacial polarization between the components of PECs, and which is adjustable in a magnetic field. In addition, it is shown that the hysteresis curves of $C_{p}$ and $R_{p}$, measured when *B* is increased and decreased, respectively, enclose a small surface area (Figure 10), with the maximum difference (at a given *B*) of about 3% (Figure 11).

When a pulsed magnetic field is used, the values of $C_{p}$ and $R_{p}$ are instantaneously induced and closely follow the shape of the magnetic field pulse (Figure 9).

These properties of the passive electrical components allow a fast adjustment of $C_{p}$ and $R_{p}$ in a wide range of values (by changing the values of $\Phi_{\mu \mathrm{Fe}}$ and *B*), thus making them very useful for various applications, including sensing, absorbing electromagnetic radiation, energy harvesting, or in human–machine interfacing.

## Figures and Tables

**Table 1:** MRSs${}_{i},i=1,2,3$ consisting of μFe and SO with masses $m_{\mu \mathrm{Fe}}$ and $m_{\mathrm{SO}}$, mass fractions $\Phi_{\mu \mathrm{Fe}}$ (wt.%), and $\Phi_{\mathrm{SO}}$ (wt.%), and with saturation magnetization $\sigma_{L_{i}}$ (Am${}^{2}$/kg).

MRSs${}_{i}$	$m_{\mu \mathrm{Fe}}$	$m_{\mathrm{SO}}$	$\Phi_{\mu \mathrm{Fe}}$ (wt.%)	$\Phi_{\mathrm{SO}}$ (wt.%)	$\sigma_{L_{i}}$ (Am${}^{2}$/kg)
MRSs${}_{1}$	0.08	3.92	2	98	0.13
MRSs${}_{2}$	0.16	3.84	4	96	0.26
MRSs${}_{3}$	0.24	3.76	6	94	0.39

## Data Availability

Experimental data are available by reasonable requests from authors. SEM, EDX, XRD and M-B magnetic measurements data for μFe were used to support this study and are available at DOI:10.1039/C9TC05687D, accessed on 18 March 2020. This prior study is cited at relevant places within the text as references.

## Abbreviations

The following abbreviations are used in this manuscript:

PECs	Passive Electrical Components
μFe	Iron Microfibers
$C_{p}$	Electrical capacitance
$R_{p}$	Electrical Resistance
MRSs	Magnetorheological Suspensions
CI	Carbonyl Iron
GB	Gauze Bandage
SO	Silicone Oil
SEMc	Scanning Electron Microscopy
EDX	Energy Dispersive X-ray

## Appendix A. Distribution of Particle Size Forming the *μ*Fe

The size distribution has been obtained from a set of SEM images at 12,000× magnification in the approximation that the maximum diameter of the particles is equal to the width of the μFe. This is a reasonable approximation since, from these images, one can see that the particles have a spherical-like shape (see Figure 2b).

## Appendix C. Experimental Data for *C*_p_ and *R*_p_ of PECs with Increasing, and Respectively Decreasing Values of *B*

**Table A1:** $C_{p}$ data (in units of nF) for PEC${}_{1}$ at measurements m${}_{i}$, with i=1,…,12 with increasing values of magnetic flux density *B* (in units of mT). The last two columns represent their average (Avg.) and the corresponding standard deviation (StDev.), respectively, and they are shown in Figure 8a and Figure 10a—black color.

*B*	m${}_{1}$	m${}_{2}$	m${}_{3}$	m${}_{4}$	m${}_{5}$	m${}_{6}$	m${}_{7}$	m${}_{8}$	m${}_{9}$	m${}_{10}$	m${}_{11}$	m${}_{12}$	Avg.	StDev.
**0**	88.00	89.23	89.41	89.58	89.76	89.94	90.11	90.29	90.46	90.64	90.82	90.99	**89.94**	**0.83**
**50**	89.50	90.75	90.93	91.11	91.29	91.47	91.65	91.83	92.01	92.19	92.36	92.54	**91.47**	**0.84**
**100**	91.00	92.27	92.46	92.64	92.82	93.00	93.18	93.37	93.55	93.73	93.91	94.10	**93.00**	**0.85**
**150**	93.00	94.30	94.49	94.67	94.86	95.05	95.23	95.42	95.60	95.79	95.98	96.16	**95.05**	**0.87**
**200**	96.00	97.34	97.54	97.73	97.92	98.11	98.30	98.50	98.69	98.88	99.07	99.26	**98.11**	**0.90**
**250**	99.00	100.39	100.58	100.78	100.98	101.18	101.38	101.57	101.77	101.07	102.17	102.37	**101.18**	**0.93**
**300**	105.00	106.47	106.68	106.89	107.10	107.31	107.52	107.73	107.94	108.15	108.36	108.57	**107.31**	**0.99**
**350**	109.00	110.53	110.75	110.96	111.18	111.39	111.62	111.83	112.05	112.27	112.49	112.71	**111.40**	**1.02**
**400**	114.00	115.60	115.82	116.05	116.28	116.51	116.74	116.96	117.19	117.42	117.65	117.88	**116.51**	**1.07**

**Table A2:** $C_{p}$ data (in units of nF) for PEC${}_{2}$ at measurements m${}_{i}$, with i=1,…,12 with increasing values of magnetic flux density *B* (in units of mT). The last two columns represent their average (Avg.) and the corresponding standard deviation (StDev.), respectively, and they are shown in Figure 8a and Figure 10a—black color.

*B*	m${}_{1}$	m${}_{2}$	m${}_{3}$	m${}_{4}$	m${}_{5}$	m${}_{6}$	m${}_{7}$	m${}_{8}$	m${}_{9}$	m${}_{10}$	m${}_{11}$	m${}_{12}$	Avg.	StDev.
**0**	74.60	75.64	74.60	75.94	76.09	76.24	76.39	76.54	76.69	76.84	76.99	77.14	**76.14**	**0.94**
**50**	75.00	76.05	75.00	76.35	76.50	76.65	76.80	76.95	77.10	77.25	77.40	77.55	**76.55**	**0.85**
**100**	77.07	78.15	77.07	78.45	78.61	78.76	78.92	79.07	79.23	79.38	79.53	79.09	**78.66**	**0.87**
**150**	80.00	81.12	80.00	81.44	81.60	81.76	81.92	82.08	82.24	82.40	82.56	82.72	**81.65**	**0.90**
**200**	82.13	83.28	82.13	83.61	83.77	83.94	84.10	84.27	84.43	84.59	84.76	84.92	**83.83**	**0.93**
**250**	86.00	87.20	86.00	87.55	87.72	87.89	88.06	88.24	88.41	88.58	88.75	88.92	**87.78**	**0.97**
**300**	88.98	90.22	88.98	90.58	90.76	90.93	91.11	91.29	91.47	91.65	91.82	92.00	**90.81**	**1.00**
**350**	92.94	93.54	92.24	93.90	94.09	94.27	94.46	94.64	94.83	95.01	95.20	95.38	**94.15**	**1.04**
**400**	100.22	101.62	100.22	102.02	102.22	102.42	102.62	102.82	103.02	103.22	103.42	103.62	**102.29**	**1.13**

**Table A3:** $C_{p}$ data (in units of nF) for PEC${}_{3}$ at measurements m${}_{i}$, with i=1,…,12 with increasing values of magnetic flux density *B* (in units of mT). The last two columns represent their average (Avg.) and the corresponding standard deviation (StDev.), respectively, and they are shown in Figure 8a and Figure 10a—black color.

*B*	m${}_{1}$	m${}_{2}$	m${}_{3}$	m${}_{4}$	m${}_{5}$	m${}_{6}$	m${}_{7}$	m${}_{8}$	m${}_{9}$	m${}_{10}$	m${}_{11}$	m${}_{12}$	Avg.	StDev.
**0**	54.60	55.36	55.47	55.58	55.69	55.80	55.91	56.02	56.13	56.24	56.35	56.46	**55.80**	**0.51**
**50**	56.00	56.78	56.90	57.01	57.12	57.23	57.34	57.46	57.57	57.68	57.79	57.90	**57.23**	**0.53**
**100**	60.07	60.91	61.03	61.15	61.27	61.39	61.51	61.63	61.75	61.87	61.99	62.01	**61.39**	**0.56**
**150**	65.00	65.91	66.04	66.17	66.30	66.43	66.56	66.69	66.82	66.95	67.08	67.21	**66.43**	**0.61**
**200**	68.30	69.25	69.39	69.52	69.06	69.80	69.93	70.07	70.21	70.34	70.48	70.62	**69.80**	**0.64**
**250**	70.00	70.98	71.12	71.26	71.40	71.54	71.68	71.82	71.96	72.10	72.24	72.38	**71.54**	**0.66**
**300**	75.28	76.33	76.48	76.63	76.78	76.93	77.08	77.23	77.38	77.53	77.68	77.83	**76.93**	**0.71**
**350**	78.00	79.09	79.25	79.40	79.56	79.72	79.07	80.03	80.19	80.34	80.50	80.65	**79.72**	**0.73**
**400**	83.22	84.38	84.55	84.71	84.88	85.05	85.21	85.38	85.55	85.71	85.88	86.05	**85.05**	**0.78**

**Table A4:** $R_{p}$ data (in units of kΩ) for PEC${}_{1}$ at measurements m${}_{i}$, with i=1,…,12 with increasing values of magnetic flux density *B* (in units of mT). The last two columns represent their average (Avg.) and the corresponding standard deviation (StDev.), respectively, and they are shown in Figure 8b and Figure 10b—black color.

*B*	m${}_{1}$	m${}_{2}$	m${}_{3}$	m${}_{4}$	m${}_{5}$	m${}_{6}$	m${}_{7}$	m${}_{8}$	m${}_{9}$	m${}_{10}$	m${}_{11}$	m${}_{12}$	Avg.	StDev.
**0**	60.65	61.50	61.62	61.74	61.86	61.98	62.10	62.22	62.35	62.47	62.59	62.71	**61.98**	**0.57**
**50**	60.69	61.54	61.66	61.79	61.91	62.03	62.15	62.27	62.39	62.51	62.64	62.76	**62.03**	**0.57**
**100**	60.69	61.54	61.66	61.79	61.91	62.03	62.15	62.27	62.39	62.51	62.64	62.76	**62.03**	**0.57**
**150**	61.52	62.38	62.50	62.62	62.75	62.87	62.99	63.15	63.24	63.36	63.48	63.61	**62.87**	**0.58**
**200**	62.41	63.29	63.41	63.54	63.66	63.79	63.91	64.04	64.16	64.29	64.41	64.54	**63.79**	**0.59**
**250**	63.42	64.31	64.44	64.57	64.69	64.82	64.95	65.07	65.20	65.33	65.45	64.59	**64.82**	**0.60**
**300**	64.25	65.15	65.28	65.41	65.54	65.67	65.79	65.92	66.05	66.18	66.31	66.44	**65.67**	**0.60**
**350**	64.83	65.74	65.87	66.00	66.13	66.26	66.39	66.52	66.65	66.78	66.91	67.04	**66.26**	**0.61**
**400**	65.33	66.25	66.38	66.51	66.64	66.77	66.90	67.03	67.16	67.29	67.42	65.55	**66.77**	**0.61**

**Table A5:** $R_{p}$ data (in units of kΩ) for PEC${}_{2}$ at measurements m${}_{i}$, with i=1,…,12 with increasing values of magnetic flux density *B* (in units of mT). The last two columns represent their average (Avg.) and the corresponding standard deviation (StDev.), respectively, and they are shown in Figure 8b and Figure 10b—black color.

*B*	m${}_{1}$	m${}_{2}$	m${}_{3}$	m${}_{4}$	m${}_{5}$	m${}_{6}$	m${}_{7}$	m${}_{8}$	m${}_{9}$	m${}_{10}$	m${}_{11}$	m${}_{12}$	Avg.	StDev.
**0**	84.43	85.61	85.78	85.95	86.12	86.28	86.45	86.62	86.79	86.96	87.13	87.30	**86.28**	**0.79**
**50**	84.00	85.18	85.34	85.51	85.68	85.85	86.62	86.18	86.35	86.52	86.69	86.86	**85.85**	**0.79**
**100**	85.95	87.16	87.33	87.50	87.67	87.84	88.02	88.19	88.36	88.53	88.70	88.88	**87.84**	**0.81**
**150**	87.00	88.22	88.39	88.57	88.74	88.91	89.09	89.26	89.44	89.61	89.78	89.96	**88.91**	**0.82**
**200**	90.42	91.68	91.87	92.05	92.23	92.41	92.59	92.77	92.95	93.13	93.31	93.49	**92.41**	**0.85**
**250**	92.00	93.29	93.47	93.66	93.84	94.02	94.21	94.39	94.58	94.76	94.94	95.13	**94.02**	**0.86**
**300**	94.48	95.80	95.99	96.18	96.37	96.56	96.75	96.94	97.13	97.31	97.50	97.69	**96.56**	**0.88**
**350**	96.00	97.34	97.54	97.73	97.92	98.11	98.30	98.50	98.69	98.88	99.07	99.26	**98.11**	**0.90**
**400**	99.88	101.28	101.48	101.68	101.88	102.08	102.28	102.48	102.68	102.88	103.08	103.28	**102.08**	**0.94**

**Table A6:** $R_{p}$ data (in units of kΩ) for PEC${}_{3}$ at measurements m${}_{i}$, with i=1,…,12 with increasing values of magnetic flux density *B* (in units of mT). The last two columns represent their average (Avg.) and the corresponding standard deviation (StDev.), respectively, and they are shown in Figure 8b and Figure 10b—black color.

*B*	m${}_{1}$	m${}_{2}$	m${}_{3}$	m${}_{4}$	m${}_{5}$	m${}_{6}$	m${}_{7}$	m${}_{8}$	m${}_{9}$	m${}_{10}$	m${}_{11}$	m${}_{12}$	Avg.	StDev.
**0**	111.43	112.99	113.21	113.43	113.66	113.88	114.10	114.32	114.55	114.77	114.99	115.22	**113.88**	**1.05**
**50**	113.90	115.49	115.72	115.95	116.18	116.40	116.63	116.86	117.09	117.31	117.54	117.77	**116.40**	**1.07**
**100**	115.12	116.73	116.96	117.19	117.42	117.65	117.88	118.11	118.34	118.58	118.81	119.04	**117.65**	**1.08**
**150**	116.66	118.29	118.52	118.76	118.99	119.22	119.46	119.69	119.92	120.16	120.39	120.62	**119.22**	**1.09**
**200**	118.92	120.58	120.82	121.06	121.30	121.54	121.77	122.01	122.25	122.49	122.72	122.96	**121.53**	**1.12**
**250**	119.56	121.23	121.47	121.71	121.95	122.19	122.43	122.69	122.91	123.15	123.36	123.63	**122.19**	**1.12**
**300**	120.48	122.17	122.41	122.65	122.89	123.13	123.37	123.61	123.85	124.09	124.34	124.58	**123.13**	**1.13**
**350**	121.34	123.05	123.28	123.53	123.77	124.01	124.25	124.50	124.74	124.98	125.23	125.47	**124.01**	**1.14**
**400**	122.88	124.60	124.85	125.09	125.34	125.59	125.83	126.08	126.32	126.57	126.81	127.06	**125.59**	**1.15**

**Table A7:** $C_{p}$ data (in units of nF) for PEC${}_{1}$ at measurements m${}_{i}$, with i=1,…,12 with decreasing values of magnetic flux density *B* (in units of mT). The last two columns represent their average (Avg.) and the corresponding standard deviation (StDev.), respectively, and they are shown in Figure 10a—black color.

*B*	m${}_{1}$	m${}_{2}$	m${}_{3}$	m${}_{4}$	m${}_{5}$	m${}_{6}$	m${}_{7}$	m${}_{8}$	m${}_{9}$	m${}_{10}$	m${}_{11}$	m${}_{12}$	Avg.	StDev.
**0**	88.40	89.64	89.81	89.99	90.35	90.52	90.70	90.88	91.05	91.23	91.41	91.58	**90.46**	**0.90**
**50**	91.34	92.62	92.80	92.99	93.35	93.53	93.72	93.90	94.08	94.26	94.45	94.63	**93.47**	**0.93**
**100**	92.79	94.08	94.27	94.46	94.82	95.01	95.20	95.38	95.57	95.75	95.94	96.13	**94.95**	**0.94**
**150**	96.00	97.34	97.54	97.73	98.11	98.30	98.50	98.69	98.88	99.07	99.26	99.46	**98.24**	**0.98**
**200**	98.00	99.37	99.57	99.76	100.16	100.35	100.55	100.74	100.94	101.14	101.33	101.53	**100.29**	**1.00**
**250**	101.76	103.18	103.38	103.59	103.99	104.20	104.40	104.61	104.81	105.01	105.22	105.42	**104.13**	**1.04**
**300**	107.00	108.50	108.71	108.93	109.35	109.57	109.78	110.00	110.21	110.42	110.64	110.85	**109.50**	**1.09**
**350**	111.00	112.55	112.78	113.00	113.44	113.66	113.87	114.11	114.33	114.55	114.77	115.00	**113.59**	**1.13**
**400**	113.80	115.39	115.62	115.85	116.30	116.53	116.76	116.99	117.21	117.47	117.67	118.00	**116.46**	**1.16**

**Table A8:** $C_{p}$ data (in units of nF) for PEC${}_{2}$ at measurements m${}_{i}$, with i=1,…,12 with decreasing values of magnetic flux density *B* (in units of mT). The last two columns represent their average (Avg.) and the corresponding standard deviation (StDev.), respectively, and they are shown in Figure 10a—black color.

*B*	m${}_{1}$	m${}_{2}$	m${}_{3}$	m${}_{4}$	m${}_{5}$	m${}_{6}$	m${}_{7}$	m${}_{8}$	m${}_{9}$	m${}_{10}$	m${}_{11}$	m${}_{12}$	Avg.	StDev.
**0**	74.60	75.64	75.79	75.94	76.09	76.24	76.39	76.54	76.69	76.84	76.99	77.14	**76.24**	**0.70**
**50**	76.21	77.28	77.43	77.59	77.74	77.89	78.04	78.20	78.35	78.50	78.65	78.81	**77.89**	**0.72**
**100**	79.11	80.21	80.37	80.53	80.69	80.85	81.01	81.16	81.32	81.48	81.64	81.80	**80.85**	**0.74**
**150**	82.45	83.61	83.77	83.94	84.10	84.27	84.43	84.60	84.76	84.93	85.09	85.26	**84.27**	**0.77**
**200**	84.18	85.35	85.52	85.69	85.86	86.03	86.20	86.37	86.53	86.70	86.87	87.04	**86.03**	**0.79**
**250**	87.54	88.77	88.94	89.12	89.29	89.47	89.64	89.82	89.99	90.17	90.34	90.52	**89.47**	**0.82**
**300**	90.42	91.69	91.87	92.05	92.23	92.41	92.59	92.77	92.96	93.14	93.32	93.50	**92.41**	**0.85**
**350**	94.98	96.31	96.50	96.69	96.88	97.07	97.26	97.45	97.64	197.83	98.02	98.21	**97.07**	**0.89**
**400**	100.22	101.62	101.82	102.02	102.22	102.42	102.62	102.82	103.02	103.22	103.42	103.63	**102.42**	**0.94**

**Table A9:** $C_{p}$ data (in units of nF) for PEC${}_{3}$ at measurements m${}_{i}$, with i=1,…,12 with decreasing values of magnetic flux density *B* (in units of mT). The last two columns represent their average (Avg.) and the corresponding standard deviation (StDev.), respectively, and they are shown in Figure 10a—black color.

*B*	m${}_{1}$	m${}_{2}$	m${}_{3}$	m${}_{4}$	m${}_{5}$	m${}_{6}$	m${}_{7}$	m${}_{8}$	m${}_{9}$	m${}_{10}$	m${}_{11}$	m${}_{12}$	Avg.	StDev.
**0**	54.60	55.37	55.47	55.58	55.69	55.80	55.91	56.02	56.13	56.24	56.35	56.46	**55.80**	**0.51**
**50**	58.00	58.81	58.93	59.04	59.16	59.28	59.59	59.51	59.62	59.74	59.86	59.97	**59.28**	**0.54**
**100**	62.11	62.98	63.10	63.22	63.35	63.47	63.60	63.72	63.85	63.97	64.09	64.22	**63.47**	**0.58**
**150**	67.00	67.94	68.07	68.21	68.34	68.47	68.61	68.74	68.88	69.01	69.14	69.28	**68.47**	**0.63**
**200**	70.18	71.16	71.30	71.44	71.58	71.72	71.86	72.00	72.14	72.28	72.42	72.56	**71.72**	**0.66**
**250**	72.00	73.01	73.15	73.30	73.44	73.58	73.73	73.87	74.02	74.16	74.30	74.45	**73.58**	**0.68**
**300**	76.88	77.96	78.11	78.26	78.42	78.57	78.73	78.88	79.03	79.19	79.34	79.49	**78.57**	**0.72**
**350**	80.00	81.12	81.28	81.44	81.60	81.76	81.92	82.08	82.24	82.40	82.56	82.72	**81.76**	**0.75**
**400**	83.22	84.38	84.55	84.71	84.88	85.05	85.21	85.38	85.55	85.71	85.88	86.05	**85.05**	**0.78**

**Table A10:** $R_{p}$ data (in units of kΩ) for PEC${}_{1}$ at measurements m${}_{i}$, with i=1,…,12 with decreasing values of magnetic flux density *B* (in units of mT). The last two columns represent their average (Avg.) and the corresponding standard deviation (StDev.), respectively, and they are shown in Figure 10b—black color.

*B*	m${}_{1}$	m${}_{2}$	m${}_{3}$	m${}_{4}$	m${}_{5}$	m${}_{6}$	m${}_{7}$	m${}_{8}$	m${}_{9}$	m${}_{10}$	m${}_{11}$	m${}_{12}$	Avg.	StDev.
**0**	60.72	61.57	61.69	61.81	62.73	62.05	62.17	62.30	62.42	62.54	62.66	62.78	**62.12**	**0.60**
**50**	59.06	59.88	60.00	60.12	62.77	60.36	60.48	60.59	60.71	60.83	60.95	61.06	**60.57**	**0.89**
**100**	59.06	59.88	60.00	60.12	62.77	60.36	60.48	60.59	60.71	60.83	60.95	61.06	**60.57**	**0.89**
**150**	59.43	60.26	60.38	60.49	63.62	60.73	60.85	60.23	61.09	61.21	61.33	62.45	**60.98**	**1.00**
**200**	60.52	61.36	61.49	61.61	64.55	61.85	61.97	62.09	62.21	62.33	62.45	64.57	**62.08**	**0.96**
**250**	61.95	62.82	62.95	63.07	65.60	63.32	63.44	63.57	63.69	63.81	63.94	65.06	**63.52**	**0.88**
**300**	63.58	64.47	64.60	64.72	66.46	64.98	65.11	65.23	65.36	65.49	65.61	66.74	**65.11**	**0.73**
**350**	64.46	65.36	65.49	65.62	67.06	65.88	66.01	66.14	66.27	66.39	66.52	66.65	**65.99**	**0.69**
**400**	65.33	66.24	66.37	66.51	67.57	66.77	66.90	67.03	67.16	67.29	67.42	67.55	**66.85**	**0.65**

**Table A11:** $R_{p}$ data (in units of kΩ) for PEC${}_{2}$ at measurements m${}_{i}$, with i=1,…,12 with decreasing values of magnetic flux density *B* (in units of mT). The last two columns represent their average (Avg.) and the corresponding standard deviation (StDev.), respectively, and they are shown in Figure 10b—black color.

*B*	m${}_{1}$	m${}_{2}$	m${}_{3}$	m${}_{4}$	m${}_{5}$	m${}_{6}$	m${}_{7}$	m${}_{8}$	m${}_{9}$	m${}_{10}$	m${}_{11}$	m${}_{12}$	Avg.	StDev.
**0**	84.43	85.61	85.78	85.95	86.16	86.29	86.45	86.62	86.79	86.96	87.13	87.30	**86.29**	**0.79**
**50**	83.00	84.16	84.33	84.49	84.66	84.23	84.99	85.16	85.32	85.49	85.66	85.82	**84.23**	**0.78**
**100**	83.67	84.84	85.01	85.17	85.34	85.51	85.68	85.84	86.01	86.18	86.35	86.51	**85.51**	**0.78**
**150**	85.50	86.70	86.87	87.04	87.21	87.38	87.55	87.72	87.89	88.07	88.24	88.41	**87.38**	**0.80**
**200**	87.95	89.18	89.36	89.54	89.71	89.89	90.06	90.24	90.42	90.59	90.77	90.94	**89.89**	**0.83**
**250**	90.79	92.06	92.24	92.42	92.61	92.79	92.97	93.15	93.33	93.51	93.69	93.88	**92.79**	**0.85**
**300**	93.56	94.87	95.05	95.24	95.43	95.62	95.80	95.99	96.18	96.36	96.55	96.74	**95.62**	**0.88**
**350**	94.00	95.32	95.50	95.69	95.88	96.07	96.27	96.44	96.63	96.82	97.01	97.20	**96.07**	**0.88**
**400**	99.88	101.28	101.48	101.68	101.88	102.08	102.28	102.48	102.68	102.88	103.08	103.28	**102.08**	**0.94**

**Table A12:** $R_{p}$ data (in units of kΩ) for PEC${}_{3}$ at measurements m${}_{i}$, with i=1,…,12 with decreasing values of magnetic flux density *B* (in units of mT). The last two columns represent their average (Avg.) and the corresponding standard deviation (StDev.), respectively, and they are shown in Figure 10b—black color.

*B*	m${}_{1}$	m${}_{2}$	m${}_{3}$	m${}_{4}$	m${}_{5}$	m${}_{6}$	m${}_{7}$	m${}_{8}$	m${}_{9}$	m${}_{10}$	m${}_{11}$	m${}_{12}$	Avg.	StDev.
**0**	111.43	112.99	113.21	113.42	113.66	113.88	114.10	114.32	114.55	114.77	114.99	115.22	**113.88**	**1.05**
**50**	112.00	113.57	113.79	114.02	114.24	114.46	114.69	114.91	115.14	115.36	115.58	115.81	**114.46**	**1.05**
**100**	114.17	115.77	115.99	116.22	116.45	116.68	116.91	117.14	117.37	117.59	117.82	118.05	**116.68**	**1.07**
**150**	115.35	116.97	117.20	117.43	117.66	117.89	118.12	118.35	118.58	118.81	119.04	119.27	**117.89**	**1.08**
**200**	116.95	118.59	118.82	119.06	119.29	119.53	119.76	119.99	120.23	120.46	120.70	120.93	**119.53**	**1.10**
**250**	117.54	119.19	119.42	119.66	119.89	120.13	120.36	120.60	120.83	121.07	121.30	121.54	**120.13**	**1.10**
**300**	118.56	120.22	120.45	120.69	120.93	121.17	121.40	121.64	121.88	122.11	122.35	122.59	**121.17**	**1.11**
**350**	119.75	121.43	121.67	121.91	122.15	122.39	122.63	122.87	123.11	123.35	123.59	123.83	**122.39**	**1.12**
**400**	122.88	124.60	124.85	125.09	125.34	125.59	125.84	126.08	126.32	126.57	126.81	127.06	**125.59**	**1.15**

## Appendix D. Average Values and Standard Deviations of *C*_p_ and *R*_p_ of PECs in Pulsed Magnetic Field

**Table A13:** Average values of $C_{p}$ data (in units of nF) and standard deviations (StDev) for PEC${}_{i},$ i=1,2,3 placed a pulsed magnetic field with amplitude B=200 mT, for time intervals: $0\;s\leq P_{1}<120\;s$, $120\;s\leq P_{2}<240\;s$, $240\;s\leq P_{3}<360\;s$, $360\;s\leq P_{4}<480\;s$ and $480\;s\leq P_{5}<600\;s$, in the format $C_{p}$/StDev.

	$P_{1}$	$P_{2}$	$P_{3}$	$P_{4}$	$P_{5}$
PEC${}_{1}$	106.00/0.02	98.11/0.17	105.80/0.19	98.25/0.09	105.90/0.14
PEC${}_{2}$	88.25/0.13	79.74/0.28	89.32/0.25	80.58/0.13	89.63/0.22
PEC${}_{3}$	68.25/0.13	59.741/0.28	69.25/0.78	60.58/0.11	69.60/0.23

**Table A14:** Average values of $R_{p}$ data (in units of kΩ) and standard deviations (StDev) for PEC${}_{i},$ i=1,2,3 placed in a pulsed magnetic field with amplitude B=200 mT, for time intervals: $0\;s\leq P_{1}<120\;s$, $120\;s\leq P_{2}<240\;s$, $240\;s\leq P_{3}<360\;s$, $360\;s\leq P_{4}<480\;s$ and $480\;s\leq P_{5}<600\;s$, in the format $R_{p}$/StDev.

	$P_{1}$	$P_{2}$	$P_{3}$	$P_{4}$	$P_{5}$
PEC${}_{1}$	60.66/0.04	55.85/0.07	55.85/0.03	55.91/0.08	61.14/0.03
PEC${}_{2}$	90.46/0.05	85.87/0.05	90.46/0.05	85.87/0.05	90.47/0.05
PEC${}_{3}$	118.46/0.05	113.87/0.05	118.46/0.05	113.87/0.05	118.47/0.05
